# DoBSeqWF: a framework for sensitive detection of individual genetic variation in pooled sequencing data

**DOI:** 10.1093/nargab/lqag021

**Published:** 2026-02-16

**Authors:** Mads Cort Nielsen, Christian Munch Hagen, Ulrik Kristoffer Stoltze, Thomas van Overeem Hansen, Mette Nyegaard, Henrik Hjalgrim, Marie Bækvad-Hansen, Anna Byrjalsen, Kjeld Schmiegelow, Karin Wadt, Jonas Bybjerg-Grauholm, Simon Rasmussen

**Affiliations:** Novo Nordisk Foundation Center for Basic Metabolic Research, Faculty of Health and Medical Sciences, University of Copenhagen, KBH N, 2200, Denmark; Department of Pediatrics and Adolescent Medicine, Rigshospitalet, 2100, KBH Ø, Denmark; Department of Congenital Disorders, Statens Serum Institute, KBH S, 2300, Denmark; Department of Clinical Genetics, Rigshospitalet, KBH Ø, 2100, Denmark; Department of Clinical Genetics, Rigshospitalet, KBH Ø, 2100, Denmark; Department of Clinical Medicine, Copenhagen University, KBH N, 2200, Denmark; Department of Congenital Disorders, Statens Serum Institute, KBH S, 2300, Denmark; Department of Clinical Medicine, Copenhagen University, KBH N, 2200, Denmark; Danish Cancer Institute, Danish Cancer Society, KBH Ø, 2100, Denmark; Department of Haematology, Rigshospitalet, KBH Ø, 2100, Denmark; Department of Congenital Disorders, Statens Serum Institute, KBH S, 2300, Denmark; Department of Clinical Genetics, Rigshospitalet, KBH Ø, 2100, Denmark; Department of Pediatrics and Adolescent Medicine, Rigshospitalet, 2100, KBH Ø, Denmark; Department of Clinical Medicine, Copenhagen University, KBH N, 2200, Denmark; Department of Clinical Genetics, Rigshospitalet, KBH Ø, 2100, Denmark; Department of Clinical Medicine, Copenhagen University, KBH N, 2200, Denmark; Department of Congenital Disorders, Statens Serum Institute, KBH S, 2300, Denmark; Novo Nordisk Foundation Center for Basic Metabolic Research, Faculty of Health and Medical Sciences, University of Copenhagen, KBH N, 2200, Denmark

## Abstract

Population screening for rare genetic diseases has the potential to increase early diagnosis and treatment, but the high cost of next-generation sequencing limits widespread implementation. Double-batched sequencing (DoBSeq) is a cost-effective method that uses two-dimensional overlapping pool sequencing to enable individual-level rare variant detection. However, the resulting high-depth, complex data require a specialized workflow for efficient, sensitive, and reproducible analysis. We developed DoBSeqWF (DoBSeq Workflow), a Nextflow-based pipeline that processes pooled sequencing data from alignment through variant calling, filtering, and final variant assignment. Using a childhood cancer cohort of 200 individuals with whole genome sequencing as a reference, we created training and validation datasets, benchmarked multiple variant callers, and implemented machine learning filters to improve rare variant detection while maintaining high sensitivity. DoBSeqWF demonstrates accurate and scalable rare variant detection within the evaluated experimental setting and provides a promising avenue for future cost-effective genetic screening programmes.

## Introduction

Rare diseases (RDs) are individually rare but accumulate to affect a large proportion of the population. Depending on the definition, as many as 10 000 RDs have been identified [1], and it is estimated that at least 6% of the European population suffers from some RD [2]. The consequences of living with an RD include delayed or misdiagnosis, incorrect treatment, prolonged hospitalization and increased mortality [3]. In addition to affecting the individual, it indirectly impacts society by increasing healthcare costs and causing loss of work ability [4]. The low prevalence and lack of identifiable biomarkers for many RDs render them unsuitable for diagnostic testing using traditional biochemical assays at scale [5].

However, at least 70% of RDs have a defined genetic etiology, and many are caused by pathogenic variants in single genes [6, 7]. This makes testing using genetic sequencing a promising approach, which can include hundreds of RDs in a single test [8]. Arguably, the high cost of sequencing has been a main factor preventing the widespread use of genetic testing for screening. In a recent pilot study, we addressed this with a novel approach termed DoBSeq (double-batched sequencing), reducing the cost of genetic testing of rare variants to a fraction compared to individual sequencing [9]. The method successfully implements pooled double sequencing, where any two pools overlap by only one individual [10, 11]. In this method, individual samples of DNA are pooled, sequenced, and analyzed twice with overlap, making it possible to pinpoint or assign unique rare variants to their unique host. In this context, pinpointable rare variants (to ensure they are carried by only one individual in a pool) are defined as variants that are private or unique to the individual within the matrix.

DoBSeq mainly reduces costs by reducing the library kit usage and the number of samples handled after the initial pooling step. Depending on the number of samples in each pool, sequencing is required to a high depth (∼2000× for 100 samples with 10 in each pool) to cover each allele for confident variant calling. The resulting pooled sequence data samples have a high allele count and depth, and in contrast to individual sequencing data, no established best practices exist for such variant analysis.

We hypothesize that the increased data complexity leads to a possible decrease in variant calling accuracy and that careful considerations should be made to choose the most suitable variant caller. Furthermore, many variant callers are designed to be lenient, requiring manual filtration, hard quality thresholds, or automated filters [12]. False positive variant calls are typically based on sequencing artifacts arising from the library preparation process or the sequencing itself and depend on both the sample preparation method and sequencing platform [13, 14]. This inherent complexity of the errors, combined with the repeated data generation with screening, makes it a suitable case for training a filtering model that improves over time as more data is generated.

Here, we present an automated workflow for analyzing DoBSeq data and a variant filtering module based on machine learning. Our pipeline implements an approach for processing overlapped pooled sequencing data, from raw reads to variant assignment. We benchmark multiple variant callers using a cohort of childhood cancer patients with available whole genome sequencing data as gold standard and develop a machine learning-based filtering module that outperforms traditional filtering methods. Our cancer case study demonstrates that the DoBSeq approach can reliably detect rare variants in batch sizes of 100 individuals while reducing sequencing costs by 80–90 percent compared to individual testing. The automated workflow enables reproducible analysis and easy adaptation as variant databases and interpretation evolve.

## Materials and methods

### Cohort

To optimize and validate the DoBSeq pipeline, we included samples from the Danish childhood cancer genomics study STAGING. The same subset and sequencing data previously described in Stoltze *et al.* [9] were used. Individuals from the cohort were divided into two separate 10 × 10 DoBSeq matrices, each containing 100 individuals, which served as a training and held-out test set for the study.

### Individual whole-genome sequencing data and variant calls

Individual whole-genome NGS data was generated from leukocytic DNA using the HiSeqX platform (Illumina, San Diego, CA, USA) with paired-end sequencing of 150bp reads with a target average coverage of 30×. Raw reads were aligned to the GRCh38 primary human reference genome sequence using BWA-MEM2 [15] (v. 2.2.1). The aligned reads were then preprocessed to remove duplicate read pairs and for base quality score recalibration using GATK [16] (v. 4.3.0) according to GATK Best Practices [17, 18]. Finally, the processed reads were subset to the genomic intervals of the 113 genes covered in Illumina’s TruSight Hereditary Cancer Panel (http://www.illumina.com/TruSightHereditaryCancer) using SAMtools v. 1.18 [19]. GATK joint genotyping was performed by first calling variants for the individual samples using HaplotypeCaller in GVCF mode. Individual VCF files were combined into multi-sample VCFs separately for the training and test sets using GenomicsDB import, and the final calls were generated using GATK GenotypeGVCF. Additionally, variants were called based on the preprocessed reads using DeepVariant [20] (v. 1.5.0) in whole-genome sequencing (WGS) mode using default settings. Using bcftools [19] (v. 1.16), the variants in the resulting VCF files were normalised by ensuring concordance of reference alleles with the reference genome, indels were left-aligned, and multi-allelic variant sites were split. No additional filtering was performed, and the intersection and symmetric difference of variant calls between GATK and DeepVariant were then defined as high and low confidence, respectively. These variant call sets were compared using RealTimeGenomics (RTG) tools vcfeval (Cleary *et al.*, 2015, unpublished software, v. 3.13).

### Overlapped pooled sequencing data

**Figure 1.**
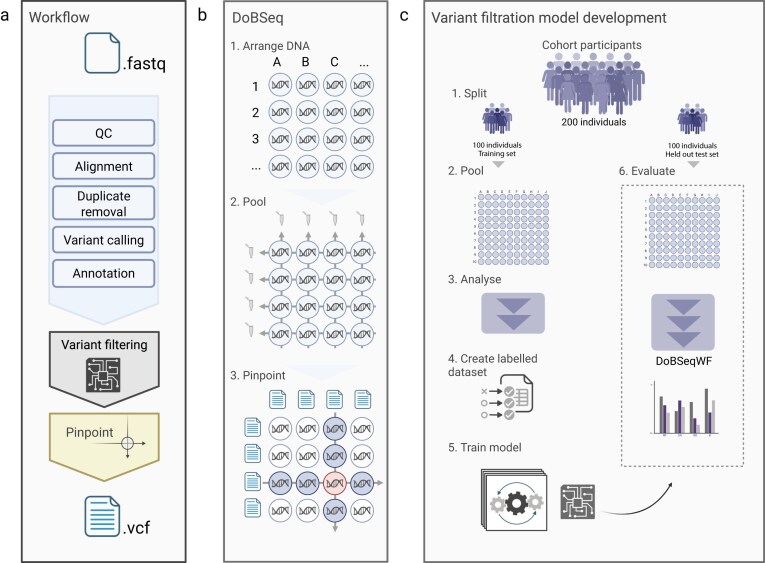
Pipeline for assigning rare variants to individuals in pooled sequencing data. **(A)** The DoBSeq workflow processes pooled sequencing data through quality control, alignment, duplicate removal, variant calling, (optional) machine learning-based filtering, and variant pinpointing, outputting VCF files with assigned variants. **(B)** The DoBSeq method: (i) DNA samples are arranged in a matrix; (ii) DNA is pooled along rows and columns and sequencing follows; (iii) Rare variants are pinpointed (assigned) to individuals by identifying variants present in exactly one row pool and one column pool, corresponding to a unique individual in the matrix. (**D**) Variant filtration model training and evaluation were performed using separate training and held-out test sets of 100 individuals each. (i) The data is split, and (ii) arranged and pooled in separate training and test matrices. Using the training matrix, (iii) technical annotations are extracted, (iv) variants are labelled based on individual sequencing data, and (v) models are trained using repeated nested cross-validation. The best-performing models are saved and tested on the held-out test dataset without retraining.

The laboratory protocol of DoBSeq is described in detail in Stoltze *et al.* [9]. In brief, for each training and test set, DNA was extracted from two 3.2-mm discs stamped from at-birth Guthrie cards for each individual. DNA concentrations were measured and normalized to ensure equimolar contributions from each sample. DoBSeq arranged the normalized DNA from the 100 individuals in a two-dimensional 10 × 10 matrix. The DNA was then pooled twice, once along each dimension, resulting in the DNA being present in exactly two pools: one along the rows and one along the columns (see Fig. 1). The 20 pools were sequenced using the TruSight Hereditary Cancer Panel, which covers 113 genes commonly associated with cancer predisposition, on the NextSeq 500 platform with a 150 bp paired-end sequencing kit and a target average coverage of 2000× per pool.

### Analysis of pooled sequencing data

Using the DoBSeq pipeline developed in this study (v. 0.2.0), the input dataset of FASTQ files containing pooled sequencing data underwent an initial quality control using FastQC (Andrews S., 2010, unpublished software, v. 0.11.8). Similar to the individual “gold standard”, the data was aligned to the GRCh38 primary human reference genome using BWA-MEM2 [21] (v. 2.2.1) and then preprocessed to remove duplicate read pairs using GATK MarkDuplicates [16] (4.6.0.0). Variant calls were generated from the processed reads using GATK Haplotypecaller [16] (4.6.0.0) in GVCF mode with allele-specific annotation output, following either individual genotyping using GATK GenotypeGVCF [16] (4.6.0.0) or with an intermediary merging of GVCF files using CombineGVCF [16] (4.6.0.0) for joint genotyping. Furthermore, three different specialized variant calling tools were used: CRISP [22] v. 0.2, LoFreq [23] v. 2.1.3.1, Octopus [24] v. 0.7.4, and NGSEP [25] v. 5.1.0. All tools were run using the correct ploidy (= 20) or pool size settings and the proper platform-specific error model if applicable. In addition to the default runs, variant calls were also generated for GATK individual genotyping without QUAL filtering by setting “-stand-call-conf” to zero, and similarly for LoFreq by removing default filters using the arguments “–no-default-filter” and increasing the the *P*-value to 1 with “–sig 1”. Similar to the gold standard calls, variants were checked, indels were left-aligned, and multi-allelic variant sites were split using bcftools norm.

### Variant pinpointing

A custom Python script was developed based on the method described in detail in Stoltze *et al.* [9] to pinpoint variants to individuals in the matrix (see Fig. 1). The algorithm iterates through the matrix one element (individual) at a time. At each step, it performs set operations to identify variants found uniquely in the current row compared to all other rows and variants found uniquely in the column compared to all other columns. The variants common between unique column and row variants were then defined as pinpointables.

### Comparison of variant calls between individual sequencing and pool sequencing data

To establish a baseline of variant calls based on gold-standard individual sequencing data for each pool in the matrix, the individual high-confidence variants from each sample in the pool were merged into a single VCF file and duplicate entries were removed using bcftools merge. This was repeated for all 20 pools. The variants called in the pooled sequencing data were then compared to this gold standard using RTG-tools vcfeval. The option “–squash-ploidy” was used to ignore the genotype of the variants, basing the evaluation only on the detection of a variant. To establish a similar baseline for pinpointable (private) variants, the high-confidence variant call set for each individual in the matrix was compared to all other individuals using bcftools isec, and only variants unique to each of the 100 individuals were retained. Again, RTG-tools vcfeval was used for comparison with the pinpointable variants from the pooled sequencing data.

### Simulation of overlapped pool sequencing data

Raw DoBSeq sequencing read data was simulated using the read generation software NGSNGS [26]. The same gene panel and reference genome were used as in the experimental setup, with genetic variation based on randomly sampled individuals from the 1000 Genomes Project [27] and NGSNGS default empirical error profile. Individuals were primarily sampled from the non-Finnish European (NFE) population. For the matrices up to 10 × 10 (100 individuals), all samples were from the NFE population. For the 24 × 24 matrix (576 individuals), the limited size of the NFE population required supplementing with individuals from other populations. No more than one related individual was included in each matrix. For each pool in the matrix, sequencing data was generated for each individual with its corresponding genetic variation at the specified coverage, and the resulting FASTQ files were then concatenated to form pools.

### Dataset and feature selection

The dataset used for developing the variant filtering model consisted of all variant calls generated by the GATK individual genotyping of pools in the training matrix. Each variant call was treated as a sample and labeled as either 0 (discordant) or 1 (concordant) based on its agreement with the low-confidence calls in the gold-standard individual call set for the specific pool. Multiple feature subsets were evaluated based on all available GATK technical annotations. One subset was created by identifying and removing highly correlated features, by calculating, clustering and plotting correlation coefficients using the R package corrplot (Wei and Simko, 2024, R package). Three additional subsets of 5, 10, and 15 features were generated using the Minimum Redundancy Maximum Relevance (MRMR) algorithm [28]. A final subset included only normally distributed features (seeSupplementary Table 1).

### Machine learning models for variant filtering

Four machine-learning approaches were evaluated for variant filtering: Gaussian Mixture Models (GMM), Random Forest (RF), Logistic Regression (LR), and Gradient Boosting (XGB). The first three were implemented using scikit-learn [29] v1.6.1, while XGB was implemented with XGBoost [30] v2.1.3. Models were trained and evaluated separately on single nucleotide variant (SNV) and insertion/deletion (indel) data using repeated nested cross-validation on the training matrix (5 repetitions, 10 outer folds, and 5 inner folds). A range of hyperparameters, as well as the different feature sets, were explored during the training and validation (see Supplementary Table 2). Based on the predicted probabilities in the training set, two prediction thresholds were established for each model: one optimizing the F1 score and another targeting 99% sensitivity. Final models were trained using the optimized hyperparameters on the full training matrix and evaluated on a separate held-out test matrix (see Fig. 1 and Supplementary Fig. 2).

### Benchmarking filtration performance

Variant filtering using GATKs variant quality score recalibration (VQSR) was performed on the jointly genotyped training dataset using a range of tranche values (90–99.9) for SNVs and indels. The best-performing tranche values, based on the F1-score, were then used for the test set. Hard threshold levels were based on the parameters defined in the GATK best practices [17, 18]. A variant would be characterized as a true positive (TP) if it was called in any of the gold standard individual WGS call sets for the specific pool and as a false positive (FP) if it was not. If a variant was found in any WGS call sets but not in the pool, it would be characterized as a false negative (FN). Performance metrics would then be estimated as follows:


\begin{eqnarray*}
{\mathrm{ Sensitivity}} = \frac{{\mathrm{ TP}}}{{\mathrm{ TP }+ \mathrm{ FN}}}
\end{eqnarray*}

\begin{eqnarray*}
\mathrm{ FDR} = \frac{{\mathrm{ FP}}}{{\mathrm{ TP} + \mathrm{ FP}}}
\end{eqnarray*}

\begin{eqnarray*}
{\mathrm{ Precision}} = \frac{{\mathrm{ TP}}}{{\mathrm{ TP} + \mathrm{ FP}}}
\end{eqnarray*}

\begin{eqnarray*}
F1 = 2\ \frac{{{\mathrm{ precision}} \cdot {\mathrm{ sensitivity}}}}{{{\mathrm{ precision}} + {\mathrm{ sensitivity}}}}
\end{eqnarray*}

For the predictions in the held-out test set, 95% confidence intervals for each metric were estimated by bootstrapping (*n* = 1000) and calculating the 2.5% and 97.5% percentiles from the resulting distributions.

## Results

### Pipeline for variant calling in overlapped pooled sequencing data

The DoBSeq workflow was developed to enable efficient, scalable, reproducible analysis of overlapped pooled sequencing data. It was implemented in Nextflow domain-specific language 2 [31]. For the initial sequence analysis steps, based upon GATK best practices for germline variant calling while introducing a reference-free filtering module and a variant assignment algorithm. The pipeline consisted of four main steps: mapping, calling, filtering, and pinpointing (Fig. 1). The input for the pipeline was a set of NGS FASTQ files and a sample table with information on matrix organization. The mapping step performs raw sequence quality control, alignment, removal of duplicate reads, and a range of optional alignment quality checks. Variant calling was done by GATK Haplotypecaller with allele-specific annotation output. The optional filtering module developed in this study, then processes and filters likely false positives in the VCF files using an SNV-specific logistic regression and an indel-specific random forest model. Variants are then assigned to each individual using the DoBSeq algorithm, which identifies unique variants in the overlapping pools, resulting in VCF files with pinpointable variants as output. The modular design allows independent execution of each step, reducing computational overhead when optimizing parameters or updating reference files. The pipeline includes optional modules for variant annotation and report generation. It can be run using conda environments or Singularity/Docker containers, and has been tested on multiple Unix-based systems. Examples of compute time and peak memory consumption for runs using simulated and experimental data are shown in Supplementary Fig. 1. The fully documented pipeline is available at https://github.com/RasmussenLab/DoBSeqWF.

### Generation of a gold standard dataset from individual WGS data

To optimize and validate the DoBSeq pipeline, we used two separate datasets, a training set and a held-out set, each composed of a 10 × 10 DoBSeq matrix of 100 individuals with WGS data available. To estimate the true genetic variation within the experiment target regions of a 113-gene panel, we used individual WGS data processed according to GATK best practices, and variants called using the two state-of-the-art germline variant callers HaplotypeCaller and DeepVariant. The outputs of the two variant callers were similar but not identical. For the training set, the callers had a concordance of 15 166 variants, which we labeled high confidence and 274 variants called by only one of them, labeled low confidence. The absolute number of low confidence variants was lower for SNVs than indels (41/233; SNVs/indels), and because of the much lower total number of indels, the low confidence accounted for 15% (233/1588, low confidence/total). The majority of low confidence indels (79%, 184/233) were called by HaplotypeCaller, indicating that DeepVariant was, in agreement with Lin *et al.* [32], more conservative. According to the DoBSeq matrix design, the variants of individuals were combined into pools by columns and rows and annotated by their theoretical ability to be pinpointed by the DoBSeq method. This aggregation resulted in a final set of 20 theoretical pools, with an average of 758 total and 72 pinpointable high-confidence variants per pool. This created a validation dataset with estimates of the true variant content and the correct individual assignments. The same analysis was done for the held-out matrix.

### Variant calling in DoBSeq overlapped pooled sequencing data

Unlike germline variant calling in individual NGS data, no well-established guidelines exist for calling variants in overlapped pooled sequencing data. To optimize the DoBSeq pipeline, we evaluated multiple variant callers on the training matrix against the high confidence validation dataset: GATK (widely used in clinical settings), LoFreq (optimized for low-frequency variants), Octopus (optimized for polyploid data), NGSEP (optimized for pool sequencing in plant screening), and CRISP (designed for pooled sequencing). Several technical limitations emerged during testing. Octopus failed to analyze two of the 20 pools due to RAM limitations (>1600 GB required), while GATK joint genotyping required manual splitting of genomic regions due to Java array length limitations. However, all callers achieved high sensitivity (0.92–0.97, see Table 1), with GATK joint genotyping showing the lowest sensitivity (0.92) and CRISP the highest (0.97). Among callers that successfully analyzed all pools, GATK and CRISP showed the highest F1 scores (0.89), indicating an optimal balance between sensitivity and false discovery rate (FDR). To explore whether reducing call confidence thresholds could further improve sensitivity, we tested lenient parameter settings for GATK and LoFreq. This approach had varying effects: GATK maintained a high F1 score (0.87) with increased sensitivity (0.96), while LoFreq’s false discovery rate increased from 0.26 to 0.73. Notably, CRISP, despite being specifically designed for pooled sequencing, achieved performance comparable to GATK (F1: 0.89). See Supplementary Table 3 for performance split by variant type. Our results indicate that specialized variant callers did not outperform GATK for pooled sequencing data, leading us to select GATK individual genotyping as our default caller.

**Table 1.** Comparison of variant callers for two-dimensional overlapping pooled sequencing data

Calling method	TP	FP	FN	Sensitivity	F1	FDR
GATK	14569	3031	711	0.953	0.886	0.172
GATK lenient	14679	3738	572	0.962	0.872	0.203
GATK joint	14026	3937	1239	0.919	0.844	0.219
CRISP	14836	3269	482	0.969	0.888	0.181
LoFreq	15018	5180	540	0.965	0.840	0.256
LoFreq lenient	15081	40883	521	0.967	0.421	0.731
NGSEP	14248	9813	1034	0.932	0.724	0.408
Octopus*	13221	1406	567	0.959	0.931	0.096

Variants were called in pools of 10 individuals in a 10 × 10 matrix using five variant callers. Variant calling was performed using lowered (lenient) confidence thresholds for GATK and LoFreq in addition to default parameters. All calls, common and rare, in all pools, were compared to the intersection of GATK and DeepVariant calls in individual WGS data. *Octopus failed to analyze 2 of the 20 pools.

### Effect of matrix size and sequencing depth on variant detection

**Figure 2.**
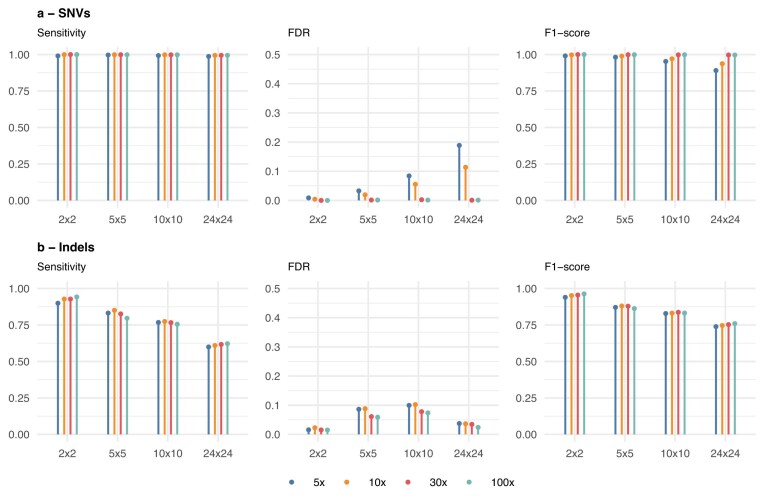
DoBSeqWF performance in detecting genetic variation in simulated overlapped pool sequencing data at various matrix sizes and allelic sequencing depths. DoBSeq data was generated with mutations from individuals from the 1000 Genomes Project, and performance was based on all variants in each pool and split by variant type **(A)** SNVs and **(B)** indels.

To evaluate the effects of matrix size and sequencing coverage on variant detection, DoBSeq data was simulated using genetic variation from individuals in the 1000 Genomes Project. Four matrix sizes (2 × 2, 5 × 5, 10 × 10, 24 × 24) and four coverage levels (5×, 10×, 30×, 100×) were evaluated. SNV sensitivity remained above 0.98 for all matrix sizes and coverages. Indel sensitivity decreased with increasing matrix size, from 0.90–0.94 at 2 × 2 to 0.60–0.62 at 24 × 24 (see Fig. 2). Coverage had a more limited effect. Sensitivity at 30× was comparable to 100× across all matrix sizes, while 5× coverage showed reduced performance. FDR for SNVs was below 0.01 at 30× coverage or higher for all matrix sizes, except 24 × 24 at 10 and 5× coverage levels, where FDR increased to 0.11–0.19. These results indicate that allelic coverage above 30× does not improve performance, and that an increased matrix size decreases assignment sensitivity for indels.

### A machine learning method for removing false positive variant calls

**Figure 3.**
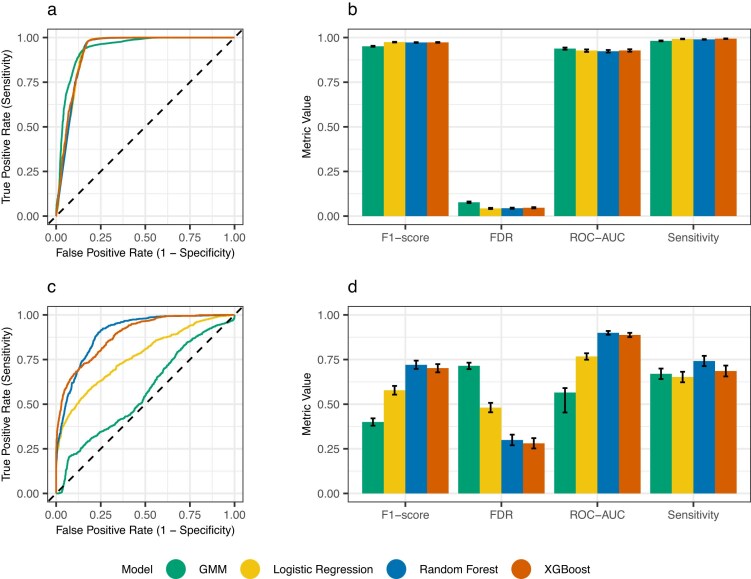
Performance of four variant filtering models in a held-out test set. (**A, B**) Performance of models trained and tested on SNVs, and (**C, D**) performance of indel models. (**A, C**) Receiver operating characteristic curves. (**B, D**) Performance metrics. Error bars indicate the 95% confidence interval calculated using bootstrapping.

Even with the best-performing variant calling method, false positive calls accounted for almost 20% in the pooled sequencing data. This high error rate confirmed the need for additional filtering to avoid false variant assignments and missed true variants due to false calls in overlapping pools. To address this challenge, we evaluated four different machine-learning approaches: Gaussian mixture models (GMM), random forest, gradient boosting, and logistic regression. Models were trained separately for SNVs and indels using technical annotations from GATK as features and validated using repeated nested 5-fold cross-validation (see Supplementary Fig. 3). Performance was assessed using F1-optimized and high-sensitivity (99%) thresholds on the held-out test matrix. All models performed well in filtering SNVs, achieving high sensitivity (0.98–0.99) and low false discovery rates (0.04–0.08) with F1-optimized thresholds (see Fig. 3). The logistic regression model slightly outperformed other approaches for SNVs with the highest F1-score of 0.974 (95% CI: 0.973–0.976) and lowest false discovery rate of 0.043 (95% CI: 0.039–0.046), while GMM showed relatively lower performance with an F1-score of 0.951 (95% CI: 0.948–0.954) and a higher FDR of 0.077 (95% CI: 0.073–0.082). All models achieved high sensitivities exceeding 0.98. However, indel filtering proved more challenging across all models, with sensitivities ranging between 0.65 (95% CI: 0.62–0.68) for logistic regression and 0.74 (95% CI: 0.71–0.77) for random forest, and widely varying false discovery rates from 0.28 (95% CI: 0.25–0.31) for XGBoost to 0.71 (95% CI: 0.70–0.73) for GMM. Based on these results, we implemented a combined approach using logistic regression for SNVs and random forest for indels in the final workflow module.

### Machine learning based filtering improved performance in the benchmark using the held-out dataset

**Figure 4.**
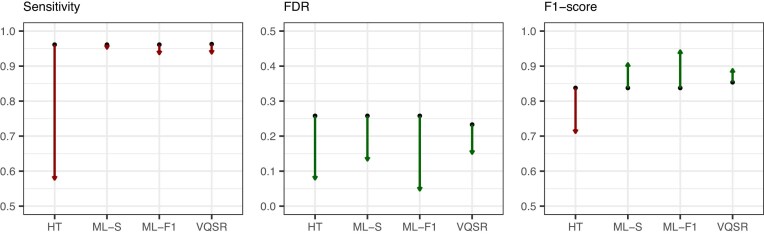
Effect of filtering approach on variant calling performance. Gain or loss of performance after filtering variant calls from GATK individual genotyping (ML-S/F1, hard filtering thresholds) or joint genotyping (VQSR) workflows.

To evaluate the final filtering module, the machine learning models, optimized for either F1 score (ML-F1) or sensitivity (ML-S), were benchmarked against GATK’s two recommended filtering methods: hard filtering thresholds and Variant Quality Score Recalibration (VQSR). We utilized unfiltered calls from GATK joint and individual genotyping workflows as a baseline. Again, the GATK joint genotyping workflow exhibited systematic failures in merging sample genotype likelihoods, requiring manual removal of 8 loci for the workflow to complete. This technical limitation rendered the joint genotyping workflow unsuitable for routine analysis of pooled data. For all variants in the test matrix, GATK individual genotyping showed similar sensitivity as joint genotyping (0.961 versus 0.963) with higher FDR (0.258 versus 0.233) (see Fig. 4). The F1-optimized ML filter achieved the most substantial FDR reduction (0.26–0.05) while maintaining high sensitivity (0.961–0.934). In comparison, hard filtering severely impacted sensitivity (dropping to 0.58), while the sensitivity-optimized ML model largely maintained its sensitivity (0.961–0.95) with an FDR reduction higher than VQSR (0.130 versus 0.150). See Supplementary Table 4 for performance split by variant type. These results demonstrate that the machine learning-based filtering approach can improve performance compared to GATK’s recommended filtering methods in pooled sequencing data, achieving FDR reduction while maintaining high sensitivity.

### Filtering had a limited positive effect on pinpointable indels in comparison to SNVs

**Figure 5.**
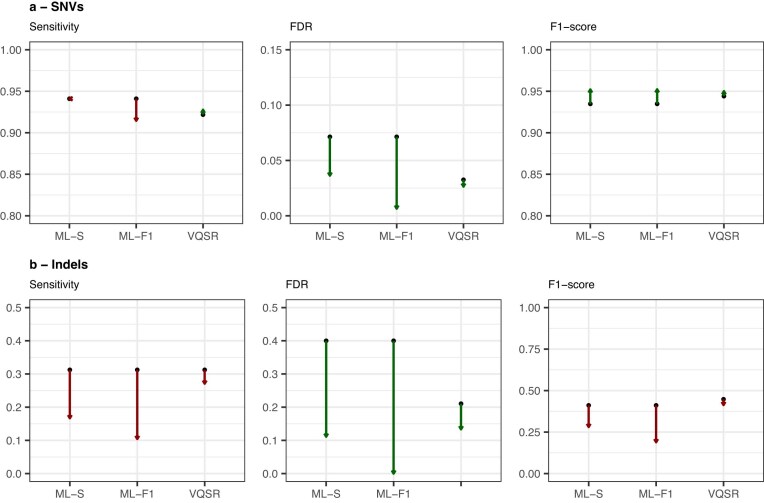
Effect of filtering approach on variant calling performance for pinpointable SNVs and indels. Gain or loss of performance after filtering pinpointable variant calls from GATK individual genotyping (ML-S/F1) or joint genotyping (VQSR) workflows. The joint genotyping workflow failed to analyse all pools. Filtering using hard thresholds was omitted due to a very large performance loss.

When evaluating overall filtering performance on pinpointable variants (Table 2), both ML-based approaches improved upon the GATK individual genotyping baseline (F1: 0.908, FDR: 0.083), with the sensitivity-optimized filter achieving F1: 0.925 (FDR: 0.037) and the F1-optimized filter achieving F1: 0.923 (FDR: 0.006). Similarly, VQSR improved the joint genotyping workflow from F1: 0.920 to 0.926. However, when separating pinpointable variants into SNVs and indels (see Fig. 5, Supplementary Table 5), it became evident that these overall improvements were driven entirely by SNV performance, while indel performance decreased with filtering.

**Table 2.** Performance of variant calling and filtering approaches on pinpointable variants

Calling method	Filtering approach	TP	FP	FN	Sensitivity	FDR	F1
GATK	No filtering	653	59	73	0.899	0.083	0.908
GATK joint*	No filtering	640	25	86	0.882	0.038	0.920
GATK	Hard threshold	3	40	723	0.004	0.930	0.008
GATK	ML - F1 optimized	625	4	101	0.861	0.006	0.923
GATK	ML - Sensitive	646	25	80	0.890	0.037	0.925
GATK joint*	VQSR	642	19	84	0.884	0.029	0.926

Performance before and after filtering variant calls from GATK individual genotyping (ML-S/F1, hard filtering thresholds) or joint genotyping (VQSR) workflows. *The joint genotyping workflow failed to analyse all pools.

For SNVs, the individual genotyping baseline F1-score was 0.935. The F1-optimized ML filter increased the score by 0.017 (to 0.952), and the sensitivity-optimized ML filter also increased it by 0.017 (to 0.952). In contrast, VQSR only increased the joint genotyping F1-score by 0.006 (from 0.944 to 0.950). For indels, the individual genotyping baseline F1-score was 0.411 with an FDR of 0.400. Neither ML filtering approach improved the F1-score, which decreased to 0.189 (ML-F1) and 0.281 (ML-S). The joint genotyping workflow achieved a higher baseline F1-score of 0.448 with an FDR of 0.211, but VQSR decreased the F1-score to 0.413.

These results indicate that the optimal variant filter in pooled sequencing data requires different strategies for SNVs and indels. For SNVs, both ML-based approaches yield a greater increase in F1-scores compared to GATK’s recommended methods. In contrast, filtering consistently decreased performance for pinpointable indels, regardless of whether ML-based or VQSR methods were used.

To assess whether variant pathogenicity affected detection performance, we investigated the detection rate stratified by predicted pathogenicity (Supplementary Table 6). The majority of gold standard variants were of unknown significance (VUS) (369/726), and of the variants found in ClinVar, the majority were benign/likely-benign (349/726). Without any filtration, the pipeline detected all eight predicted loss-of-function or known pathogenic variants with one false positive assignment, indicating that detection performance is consistent regardless of variant pathogenicity.

## Discussion

Here we present an automated workflow for analyzing and assigning rare variants to individuals in overlapping pooled sequencing data. Using a Danish childhood cancer cohort with known genetic variation, we benchmarked multiple variant callers in the workflow and found a generally high sensitivity accompanied by high FDR. Based on GATK technical annotations, we developed a machine learning based variant filter module. A benchmark compared to hard filtering thresholds and GATK VQSR revealed higher F1 scores for the module as well as a general reduction of FDR while maintaining high sensitivities. The effect of the filter on individually pinpointed variants was less definitive, with an increasing F1 score for SNV calls but no improvement for indels.

The reported performance of variant callers in low frequency and pool-seq data is inconsistent across studies, with callers showing highly variable performances [33–35]. This inconsistency likely stems from differences in parameter optimization and the varying use of synthetic or real experimental data. Nevertheless, the slightly superior performance of GATK in our study is consistent with in-silico results reported by Huang *et al.* [35]. Furthermore, we find that CRISP did not outperform other callers in our study, contrary to previous findings [36] and its use in multiple pool-seq studies [37–39]. Although resource usage was not a primary focus of our comparison, it is noteworthy that both GATK joint genotyping workflow and Octopus failed to analyze the data successfully. In contrast, CRISP executed rapidly, which could be advantageous for larger matrix sizes.

Very few studies have investigated the performance of variant calling in overlapped pooled sequencing data, with experimental data validated against individual sequencing. One such study [10], which employed a design similar to DoBSeq, randomly sampled 60 variants in a 12 × 12 matrix of individuals and verified these by individual sequencing. This approach did not account for false negative calls, making it impossible to determine the sensitivity of their method. Nevertheless, they reported FDRs of 0.1 and 0.2 for all callable and pinpointable variants, respectively. These results are converse to the FDRs of 0.2 and 0.1 observed with raw calls in our workflow. In our case, the FDR decreased, as expected, when assessing only pinpointed variants since the algorithm inherently applies additional filtering by requiring a variant to be detected in two pools. The accompanying decrease in sensitivity for pinpointable variant calls is likely attributable to their lower allelic frequency in the pools. Most variants in the call set are shared among multiple individuals in each pool, resulting in higher frequencies, making them readily detectable with a mean coverage of >3000× in most pools. Nonetheless, compared to the study above, our method achieved superior FDRs of 0.05 and 0.01 after applying machine learning filtering.

The binary classification task of filtering true and false variant calls in our analysis proved substantially easier for SNVs than indels. All model types performed strongly in filtering SNVs, achieving high sensitivity (0.98–0.99) and low false discovery rates (0.04–0.08) with F1-optimized thresholds. In contrast, indel filtering sensitivities ranged from 0.65 to 0.74, and FDRs from 0.3 to 0.74. This difference is likely partly caused by the much smaller indel training dataset (2126 versus 12761) with significantly lower confidence in its labeling. In the gold standard dataset, 32% (685/2126) of indels were called by only one of the two callers (GATK or DeepVariant), a discordance that aligns with findings from other studies [32, 40]. Such a smaller and noisier dataset inherently creates worse conditions for the models to learn patterns in the technical annotations and generalize effectively.

Another reason for the differential challenge of classifying SNVs and indel errors is likely their different origins. False positive SNVs often arise due to substitutions during sequencing and incorrect alignment [14, 41]. Both cases should theoretically produce discriminatory quality metrics that can be addressed through filtering models like the one developed here. Although overlapping, the primary source is likely slightly different for indels. In a study by Li *et al.* [14], ow-complexity regions (LCRs) were shown to harbor 80%–90% of indel errors despite comprising only 2% of the genome. They identify two main sources of errors in these regions: PCR amplification artifacts, often along homopolymer runs, and sub-optimal alignments in LCRs that are not efficiently resolved, even with local-reassembly-based variant callers. The discordance in indel calls between GATK and DeepVariant likely stems from these sub-optimal alignments, as GATK performs local reassembly while DeepVariant does not. These errors should, however, be discernible through quality metrics and, therefore, filterable. On the other hand, PCR amplification errors are much more challenging to detect since they occur before sequencing and consequently appear indistinguishable from true mutations, though possibly at lower frequencies. Rather than addressing this specific type of error computationally, a more direct biochemical approach might be more effective, as reviewed by Salk *et al.* [42].

The primary motivation for pooled sequencing is cost reduction, acknowledging that the sensitivity will never exceed individual sequencing. While the two-dimensional pooling strategy may not minimize the number of tests per positive sample as effectively as other methods, it provides important advantages. A notable alternative approach relies on hierarchical pooling, initially using large pools and then creating progressively smaller pools from positive results [11]. This method has the potential to reduce the sequencing costs even further. However, hierarchical pooling requires precise knowledge of the target variants at the time of analysis, as subsequent laboratory steps depend on initial findings. The primary advantage of the two-dimensional pooling strategy is that all rare variants are assigned in a single step. Many disease-relevant variants are of unknown significance, but their interpretation is likely to evolve with ongoing research. Our method allows variant assignment data to be easily re-analyzed against updated databases. This is particularly straightforward using an automated pipeline, where modules can be easily modified and steps rerun. Similarly, updating the workflow and rerunning the entire sequence analysis remains uncomplicated as new NGS analysis tools and variant callers are developed.

Using WGS data as a benchmark comes with certain limitations. Based on the results by Sun *et al.* [43], 30× WGS is likely to achieve >99% sensitivity and positive predictive value (PPV) for both homozygous and heterozygous SNVs. However, the sensitivity for indels will be lower and likely below 90% for both homozygous and heterozygous indels, with a PPV below 85% for heterozygous indels. These accuracy limitations will arguably impact the validation process, as false-positive WGS calls may be incorrectly labeled as false negatives in the variant calls from DoBSeq data, and false-negative WGS calls may lead to incorrectly labeled false positives. This challenge could be addressed by increasing WGS sequencing depth, incorporating long-read sequencing technologies, or using DNA from well-characterized reference genomes as part of the matrices.

The current implementation of DoBSeqWF has several limitations. The experimental evaluation is based on a limited cohort size and a single matrix configuration, which restricts statistical power and limits generalization of performance across rare genetic conditions and experimental regimes. The method resolves and assigns only variants that are private to single individuals within a matrix; variants shared by two or more individuals cannot be uniquely assigned and require individual follow-up sequencing. As matrix size increases, the proportion of private variants decreases, thereby reducing sensitivity and potentially affecting the detection and assignment of more common pathogenic variants, including founder variants. The workflow does not currently perform individual genotyping and is limited to single-nucleotide variants and small insertions and deletions. In addition, the validation presented here is based on a childhood cancer cohort using a cancer predisposition gene panel. Performance characteristics may differ for other diseases, genetic backgrounds, or target panels, and extending the method to these contexts would require further validation.

In conclusion, DoBSeqWF provides an automated and reproducible workflow for the analysis of two-dimensional pooled sequencing data, including machine learning-based variant filtering and the assignment of rare genetic variants to individuals. Using pooled and individual sequencing data, we demonstrate that accurate low-cost rare variant detection and assignment is feasible within the evaluated experimental setting.

## Supplementary Material

lqag021_Supplemental_File

## Data Availability

The DoBSeq workflow is publicly available at https://github.com/RasmussenLab/DoBSeqWF and https://doi.org/10.5281/zenodo.15632396, and the machine learning filter models, along with scripts for reproducing data analyses and figures, can be accessed at https://github.com/madscort/dwf-filter/ and https://doi.org/10.5281/zenodo.15632353. The underlying sequence data cannot be shared publicly due to the privacy of individuals who participated in the study and restrictions from the ethical approval. The data will be shared on reasonable request to the corresponding author, subject to appropriate ethical and legal approvals.
