# Supplementary material for: DoBSeqWF: a framework for sensitive detection of individual genetic variation in pooled sequencing data
Source: NAR Genom Bioinform. 2026 Feb 16;8(1):lqag021. doi: 10.1093/nargab/lqag021 (PMC12907731; doi:10.1093/nargab/lqag021)
Supplement: lqag021_Supplemental_File [file lqag021_supplemental_file.pdf]

SUPPLEMENTARY MATERIAL

DoBSeqWF: A framework for sensitive detection of individual genetic variation in pooled sequencing data

AUTHORS

Mads Cort Nielsen[1,2], Christian Munch Hagen[4], Ulrik Kristoffer Stoltze[3], Thomas van Overeem Hansen[3,6], Mette Nyegaard[4], Henrik Hjalgrim[5,6,7], Marie Bækvad-Hansen[4], Anna Byrjalsen[3], Kjeld Schmiegelow[2,6], Karin Wadt[3,6], Jonas Bybjerg-Grauholm[4], Simon Rasmussen[1]*

[1] Novo Nordisk Foundation Center for Basic Metabolic Research, Faculty of Health and Medical Sciences, University of Copenhagen, KBH N, 2200, Denmark

[2] Department of Pediatrics and Adolescent Medicine, Rigshospitalet, 2100, KBH Ø, Denmark

[3] Department of Clinical Genetics, Rigshospitalet, KBH Ø, 2100, Denmark

[4] Department of Congenital Disorders, Statens Serum Institute, KBH S, 2300, Denmark

[5] Danish Cancer Institute, Danish Cancer Society, KBH Ø, 2100, Denmark

[6] Department of Clinical Medicine, Copenhagen University, KBH N, 2200, Denmark

[7] Department of Haematology, Rigshospitalet, KBH Ø, 2100, Denmark

* To whom correspondence should be addressed. Tel: +45 35 33 21 59; Email: srasmuss@sund.ku.dk

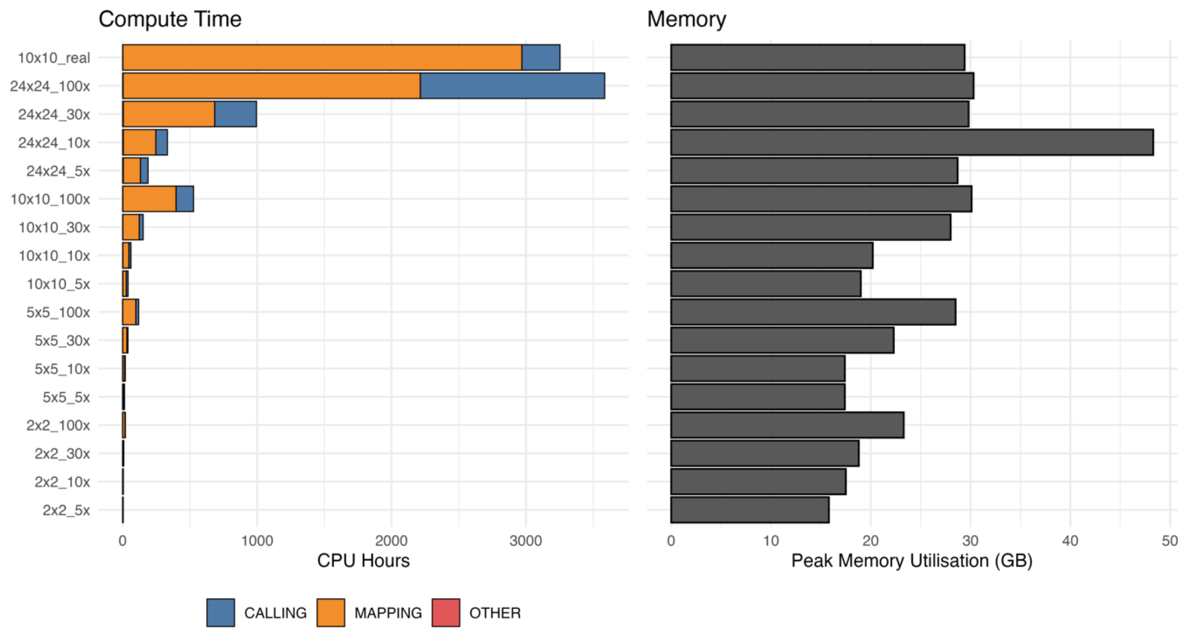

Supplementary Figure 1. Runtime and peak memory usage for DoBSeqWF at various matrix sizes and allelic coverages. Compute time is estimated based on job duration, CPU allocation and average CPU utilization.

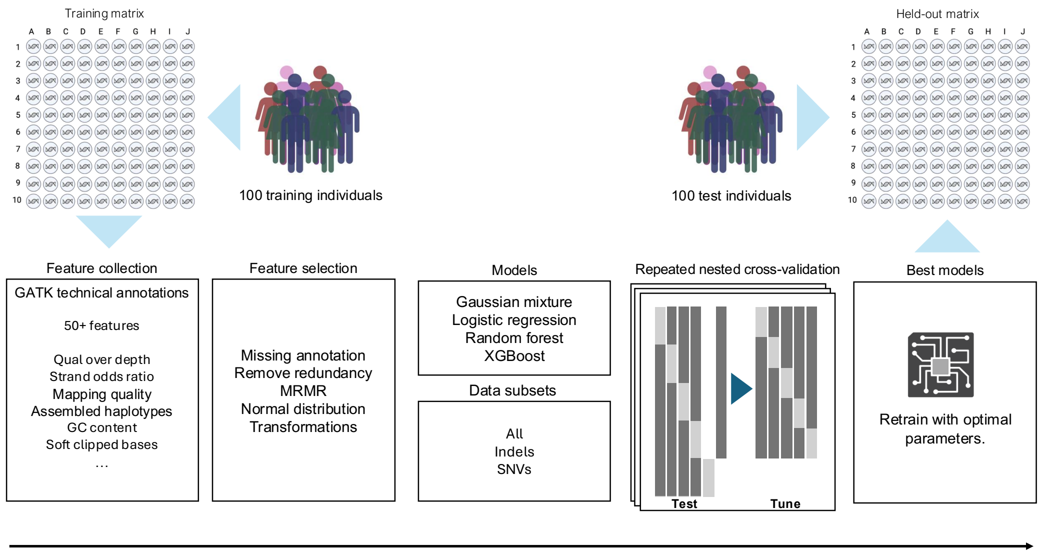

Supplementary Figure 2. Filter model training overview. The models are trained and tested on separate 10x10 DoBSeq matrices each including data from 100 individuals. All feature selection, hyperparameter tuning and final model selection is done using the variant data from the training set. The final prediction results and benchmark is performed with saved model weights on the full held-out test set.

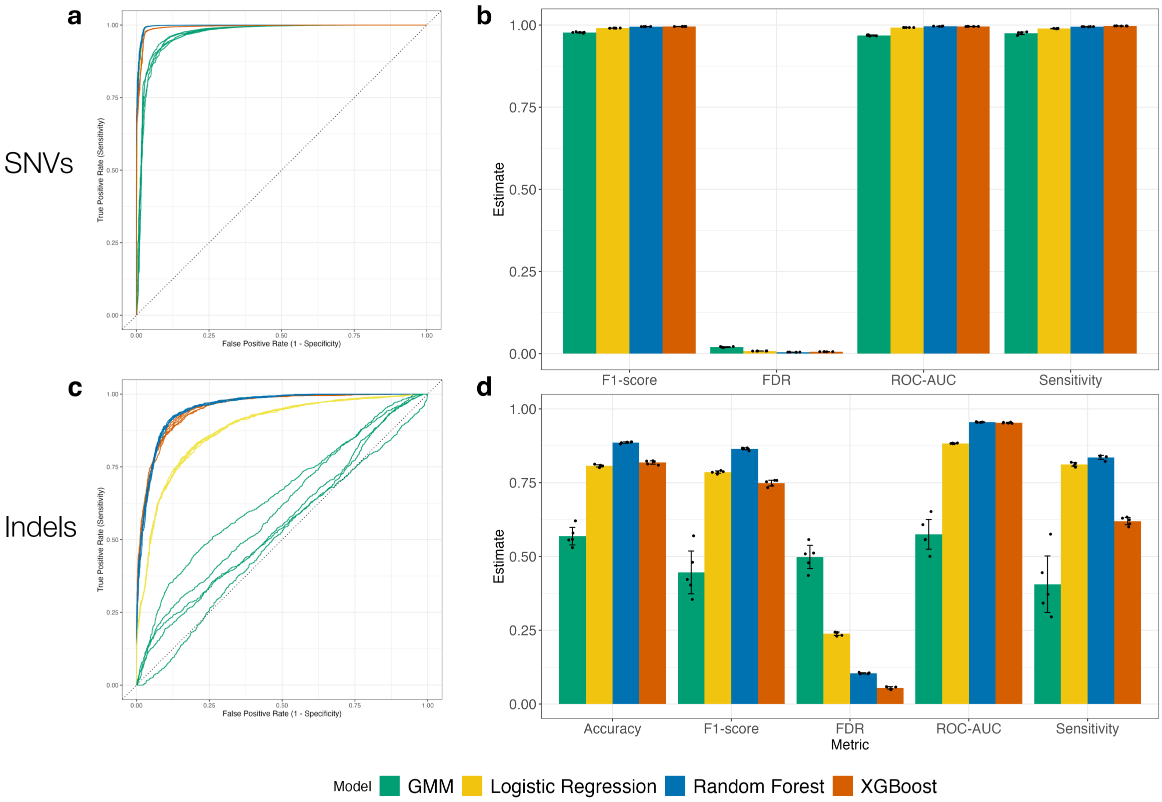

Supplementary Figure 3. Performance of four variant filtering models during training using repeated nested cross validation. (**a-b**) Performance of models trained and tested on SNVs, and (**c-d**) performance of indel models. (**a,c**) ROC curves with one line per repetition. (**b,d**) Performance metrics. Height indicates the mean over all repetitions, points indicate result per repetition and error bars indicate the 95% confidence interval of the mean.

Type	Feature ID	Description
Site-specific	GQ	Genotype quality
Site-specific	DP	Approximate read depth; some reads may have been filtered
Site-specific	ASSEMBLED_HAPS	Haplotypes detected by the assembly region before haplotype filtering is applied
Site-specific	HAPCOMP	Edit distances of each alt allele's most common supporting haplotype from closest germline haplotype, excluding differences at the site in question
Site-specific	HAPDOM	For each alt allele, fraction of read support that best fits the most-supported haplotype containing the allele
Site-specific	HEC	Counts of support for haplotype groups excluding difference at the site in question
Site-specific	MLEAC	Maximum likelihood expectation (MLE) for the allele counts (not necessarily the same as the AC), for each ALT allele, in the same order as listed
Site-specific	MLEAF	Maximum likelihood expectation (MLE) for the allele frequency (not necessarily the same as the AF), for each ALT allele, in the same order as listed
Site-specific	X_GCC	Flow: percentage of G or C in the window around hmer
Allele-specific	AF	Allele Frequency, for each ALT allele, in the same order as listed
Allele-specific	AD	Allelic depths for the ref and alt alleles in the order liste
Allele-specific	BaseQRankSum	Allele specific Z-score from Wilcoxon rank sum test of each Alt Vs. Ref base qualities]
Allele-specific	FS	Phred-scaled p-value using Fisher's exact test to detect strand bias
Allele-specific	MQ	RMS Mapping Quality
Allele-specific	MQRankSum	Z-score From Wilcoxon rank sum test of Alt vs. Ref read mapping qualities
Allele-specific	QD	Variant Confidence/Quality by Depth
Allele-specific	ReadPosRankSum	Z-score from Wilcoxon rank sum test of Alt vs. Ref read position bias
Allele-specific	SOR	Symmetric Odds Ratio of 2x2 contingency table to detect strand bias
Allele-specific	ClippingRankSum	Z-score From Wilcoxon rank sum test of Alt vs. Ref number of hard clipped bases
Allele-specific	LikelihoodRankSum	Z-score from Wilcoxon rank sum test of Alt Vs. Ref haplotype likelihoods

Supplementary Table 1. Description of technical annotations used for the final filtering models as given by GATK. Annotations are either site or variant allele specific.

Model Type	Parameter	SNV range	Indel range
GMM	n_components	2-8	2-8
GMM	covariance_type	full, tied	full, tied
Logistic Regression	C	0.00001-100	0.00001-500
Logistic Regression	penalty	l1, l2, elasticnet	l1, l2, elasticnet
Logistic Regression	class_weight	None, balanced	None, balanced
Random Forest	n_estimators	50-500	5-250
Random Forest	max_depth	5-30	5-30
Random Forest	min_samples_leaf	2-10	2-10
Random Forest	class_weight	None, balanced	None, balanced
XGBoost	n_estimators	10-300	10-300
XGBoost	max_depth	3-10	3-10
XGBoost	learning_rate	0.01-0.9	0.01-0.9
XGBoost	colsample_bytree	0.6-1.0	0.6-1.0
XGBoost	class_weight	None, balanced	None, balanced

Supplementary Table 2. Hyperparameter search ranges for the model types used in the study.

Calling method	Variant type	TP	FP	FN	Sensitivity	Precision	F1	FDR
CRISP	INDEL	1025	1229	440	0.700	0.455	0.551	0.545
CRISP	SNV	13811	2040	42	0.997	0.871	0.930	0.129
GATK	INDEL	833	1821	621	0.573	0.314	0.406	0.686
GATK	SNV	13736	1210	90	0.993	0.919	0.955	0.081
GATK joint	INDEL	1010	2423	429	0.702	0.294	0.415	0.706
GATK joint	SNV	13016	1514	810	0.941	0.896	0.918	0.104
GATK lenient	INDEL	931	2069	491	0.655	0.310	0.421	0.690
GATK lenient	SNV	13748	1669	81	0.994	0.892	0.940	0.108
LoFreq	INDEL	1312	2244	401	0.766	0.369	0.498	0.631
LoFreq	SNV	13706	2936	139	0.990	0.824	0.899	0.176
LoFreq lenient	INDEL	1366	2546	393	0.777	0.349	0.482	0.651
LoFreq lenient	SNV	13715	38337	128	0.991	0.263	0.416	0.737
NGSEP	INDEL	529	653	914	0.367	0.448	0.403	0.552
NGSEP	SNV	13716	9124	120	0.991	0.601	0.748	0.399
Octopus*	INDEL	950	1149	382	0.713	0.453	0.554	0.547
Octopus*	SNV	12269	256	185	0.985	0.980	0.982	0.020

Supplementary Table 3. Comparison of variant callers for two-dimensional overlapping pooled sequencing data split by variant types. Variants were called in pools of 10 individuals in a 10x10 matrix using five variant callers. Variant calling was performed using lowered (lenient) confidence thresholds for GATK and LoFreq in addition to default parameters. All calls, common and rare, in all pools, were compared to the intersection of GATK and DeepVariant calls in individual WGS data. *Octopus failed to analyze two of the 20 pools.

Calling method	Filtering approach	Variant type	TP	FP	FN	Sensitivity	F1	FDR
GATK	HT	INDEL	494	611	855	0.366	0.403	0.553
GATK	HT	SNV	8309	119	5620	0.597	0.743	0.014
GATK joint*	No filtering	INDEL	967	2363	400	0.707	0.412	0.710
GATK joint*	No filtering	SNV	13759	2108	170	0.988	0.924	0.133
GATK	ML - F1	INDEL	599	359	754	0.443	0.518	0.375
GATK	ML - F1	SNV	13680	317	249	0.982	0.980	0.023
GATK	ML - Sensitive	INDEL	726	546	617	0.541	0.555	0.429
GATK	ML - Sensitive	SNV	13784	1635	145	0.990	0.939	0.106
GATK	No filtering	INDEL	893	2109	450	0.665	0.411	0.703
GATK	No filtering	SNV	13786	2991	143	0.990	0.898	0.178
GATK joint*	VQSR	INDEL	561	1274	799	0.413	0.351	0.694
GATK joint*	VQSR	SNV	13750	1250	179	0.987	0.951	0.083

Supplementary Table 4. Performance of variant calling and filtering approaches on all variants in pools split by variant type. Performance before and after filtering variant calls from GATK individual genotyping (ML-S/F1, hard filtering thresholds) or joint genotyping (VQSR) workflows. *The joint genotyping workflow failed to analyse all pools.

Calling method	Filtering approach	Variant type	TP	FP	FN	Sensitivity	F1	FDR
GATK	HT	INDEL	0	6	48	0.000	0.000	1.000
GATK	HT	SNV	3	34	675	0.004	0.008	0.919
GATK joint*	No filtering	INDEL	15	4	33	0.313	0.448	0.211
GATK joint*	No filtering	SNV	625	21	53	0.922	0.944	0.033
GATK	ML - F1	INDEL	5	0	43	0.104	0.189	0.000
GATK	ML - F1	SNV	620	4	58	0.914	0.952	0.006
GATK	ML - Sensitive	INDEL	8	1	40	0.167	0.281	0.111
GATK	ML - Sensitive	SNV	638	24	40	0.941	0.952	0.036
GATK	No filtering	INDEL	15	10	33	0.313	0.411	0.400
GATK	No filtering	SNV	638	49	40	0.941	0.935	0.071
GATK joint*	VQSR	INDEL	13	2	35	0.271	0.413	0.133
GATK joint*	VQSR	SNV	629	17	49	0.928	0.950	0.026

Supplementary Table 5. Performance of variant calling and filtering approaches on pinpointable variants split by variant type. Performance before and after filtering variant calls from GATK individual genotyping (ML-S/F1, hard filtering thresholds) or joint genotyping (VQSR) workflows. *The joint genotyping workflow failed to analyze all pools.

Classification	TP	FP	FN	Sensitivity	F1	FDR
B	86	1	24	0.782	0.873	0.011
LB	216	8	23	0.904	0.933	0.036
VUS	343	49	26	0.930	0.901	0.125
pLoF/P	8	1	0	1.000	0.941	0.111

Supplementary Table 6. Performance of DoBSeqWF with GATK individual genotyping without filtration stratified by ClinVar classification and predicted loss-of-function. Classification is based on ClinVar annotation, with pathogenic variants merged with variants predicted as loss-of-function with high confidence using LOFTEE. Variants absent from ClinVar were classified as VUS.
